# Serum CXCL13 as a Novel Biomarker in Oral Squamous Cell Carcinoma

**DOI:** 10.1002/cam4.70263

**Published:** 2024-09-30

**Authors:** Shin Tojo, Koh‐ichi Nakashiro, Nobuyuki Kuribayashi, Daisuke Uchida

**Affiliations:** ^1^ Department of Oral and Maxillofacial Surgery Ehime University Graduate School of Medicine Toon Japan

**Keywords:** biomarker, C‐X‐C motif chemokine ligand 13, oral squamous cell carcinoma, prognosis

## Abstract

**Background:**

Despite its low sensitivity (approximately 30%), squamous cell carcinoma (SCC) antigen is commonly utilized as a serum tumor marker for oral SCC (OSCC) in clinical settings. The objective of this research was to identify novel biomarkers for OSCC.

**Methods:**

Initially, we performed microarray analysis to evaluate the gene expression signatures of primary OSCC and normal oral mucosal tissues. Our findings showed the C‐X‐C motif chemokine ligand 13 (CXCL13) to be a promising novel biomarker as it was consistently overexpressed in primary OSCC tissues, a conclusion corroborated by polymerase chain reaction results. Subsequently, we measured serum CXCL13 levels in 125 patients with OSCC using a sandwich enzyme‐linked immunosorbent assay and compared the results with those of 29 healthy individuals.

**Results:**

Remarkably, the levels of serum CXCL13 were consistently elevated in patients with OSCC, and the high expression of serum CXCL13 was notably associated with tumor size and neck lymph node metastasis. Patients with advanced OSCC with high‐serum CXCL13 levels exhibited poor prognosis regarding both overall and disease‐free survival. Finally, spatial transcriptome analysis revealed CXCL13 and CD8 expressions within tumor area clusters but not in adjacent normal areas, suggesting specific overexpression of CXCL13 in primary OSCC tissues.

**Conclusion:**

These findings imply that serum CXCL13 holds diagnostic and prognostic value, showing promise as a novel biomarker for OSCC.

## Graphical abstract

Serum CXCL13 is a potential novel biomarker with diagnostic and prognostic significance for oral squamous cell carcinoma.

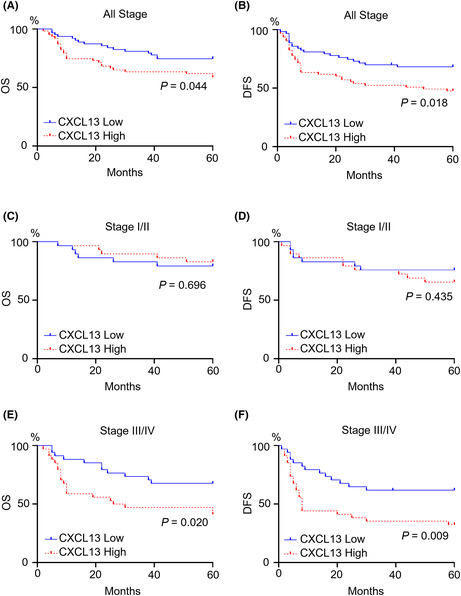

## 1 Introduction

Oral cancer encompasses malignant epithelial tumors that develop in various areas, such as the buccal mucosa, upper and lower gingiva, hard palate, tongue, and floor of the mouth. Globally, there were about 370,000 cases of oral and pharyngeal cancer in 2020, with a projected consistent rise in new cases annually [1]. Treatment options for oral cancer typically involve surgical procedures, radiotherapy, and chemotherapy, with an increasing tendency toward multidisciplinary approaches. Recent advancements have introduced molecular targeted therapies and immune checkpoint inhibitors as viable treatment options [2, 3]. However, despite these advancements, the survival rate of patients with oral cancer has seen limited improvement, primarily attributed to delayed early detection and resistance to conventional treatments [4]. Squamous cell carcinoma (SCC) makes up about 90% of oral cancer cases, originating from mucosal epithelium [1]. The survival rate for patients with oral squamous cell carcinoma (OSCC) over a period of 5 years remains around 50%, with 25%–50% of patients experiencing local or regional recurrence and distant metastasis after primary treatment [5, 6]. Hence, early identification of OSCC is essential for enhancing survival rates through timely interventions. To achieve this goal, there is a critical need to identify reliable diagnostic and prognostic biomarkers specific to OSCC. While several studies have reported potential biomarkers for OSCC, only a few of these biomarkers have been confirmed and effectively implemented in clinical settings. Specifically, the SCC antigen is one of the most recognized tumor markers for OSCC; however, its sensitivity is limited (around 30%), particularly in early‐stage cancers [7]. In recent years, various biomarkers, such as microRNA‐196a‐5p for predicting neck lymph node metastasis (LNM) [8] and FK506‐binding protein 51 protein for prognosis prediction [9], have been identified. However, their utility for early detection remains limited, and precise diagnostic biomarkers for OSCC remain elusive. In a previous study, we employed microarray analysis to compare the gene expression signatures of primary OSCC and normal oral mucosa tissues [10]. Our results showed nine genes exhibiting over 20‐fold higher expression in primary OSCC tissues. Among these genes, we specifically focused on C‐X‐C motif chemokine ligand 13 (CXCL13) as a novel biomarker candidate for OSCC. CXCL13 plays a pivotal role in immune response regulation, particularly in the recruitment, activation, and regulation of adaptive immune cells. Its interaction with the C‐X‐C chemokine receptor type 5 (CXCR5) is essential for the development of tertiary lymphoid structures (TLS), which are well‐organized clusters of T cells, B cells, and dendritic cells [11]. Moreover, elevated CXCL13 expression levels have been observed in several types of cancers, such as breast, prostate, and gastrointestinal cancers [12]. Additionally, since CXCL13 is a gene encoding a soluble secreted protein, we posit that it holds potential for use as a serum tumor marker. Consequently, this study was designed to measure the serum levels of CXCL13 in patients with both early and advanced OSCC, as well as in healthy individuals, to evaluate its utility as a biomarker for OSCC detection. Furthermore, we investigated the relationship between serum CXCL13 levels and overall survival (OS) as well as disease‐free survival (DFS) in patients with OSCC, considering its potential to predict prognosis.

## 2 Methods

### 2.1 Patients and Characteristics

We enrolled 125 patients diagnosed with OSCC who underwent radical treatment at the Department of Dental, Oral Surgery, and Orthodontics, Ehime University Hospital, between May 2004 and March 2012. Clinical staging and grading were determined based on the Union for International Cancer Control TNM Classification of Malignant Tumors 7th Edition and the World Health Organization criteria (Table S1). The exclusion criteria encompassed patients with concurrent malignancies and those without SCC antigen testing. The control group comprised 29 healthy individuals; their serum samples were utilized for comparisons. The time frame for observation was initiated from the day of sample collection until the final visit conducted during the investigation period. Ethical approval for this study was granted by the Ethics Review Board of Ehime University Hospital (Approval Number: 1002011).

### 2.2 Total RNA Extraction

The extraction of total RNA was carried out using a TissueLyser (Qiagen, Hilden, Germany) to homogenize tissues and ISOGEN (Nippon Gene, Tokyo, Japan) to lyse them, following the manufacturer's instructions. The concentrations of the extracted RNA were determined using an ultraviolet spectrophotometer (GeneQuant; GE Healthcare Bioscience, Piscataway, NJ, USA). To confirm the integrity of the RNA samples, the Agilent 2100 Bioanalyzer (Agilent Technologies, Santa Clara, CA, USA) was employed, and the samples were stored at −80°C until further experimentation.

### 2.3 Microarray

Total RNA was extracted from tissue samples, comprising 10 primary OSCC and three normal oral mucosa tissue. One microgram of total RNA was employed to synthesize double‐stranded complementary DNA (cDNA), followed by transcription with digoxigenin‐labeled nucleotides (Roche Diagnostics, Basel, Switzerland) and fragmentation using an Applied Biosystems Chemiluminescent RT‐IVT Labeling Kit (Thermo Fisher Scientific, Waltham, MA, USA). The resulting fragments were then hybridized into an Applied Biosystems Human Genome Survey Microarray (version 2.0, Thermo Fisher Scientific). Following sample washing, the Chemiluminescent Detection Kit (Thermo Fisher Scientific) was employed, and the processed arrays were then visualized by an Applied Biosystems 1700 Chemiluminescent Microarray Analyzer (Thermo Fisher Scientific). The results were normalized using GeneSpring GX 14 (Agilent Technologies) and then uploaded to the Gene Expression Omnibus (GEO, http://www.ncbi.nlm.nih.gov/geo; experiment number GSE36090) following the Minimum Information About Microarray Experiment (MIAME) guidelines [10].

### 2.4 RT‐Quantitative PCR (RT‐qPCR)

To synthesize cDNA, we employed 1 μg of total RNA and used SuperScript IV VILO Master Mix (Thermo Fisher Scientific) to reverse transcribe it, according to the manufacturer's instructions. The expression levels of CXCL13 mRNA were determined using the threshold cycle (Ct) with the TaqMan PCR system (Thermo Fisher Scientific), utilizing hydroxymethylbilane synthase (HMBS) as an internal control. PCR amplification was conducted in a final reaction volume of 20 μL, comprising 10 μL of TaqMan Universal Master Mix II (Thermo Fisher Scientific), 1 μL of TaqMan probe and primers (Thermo Fisher Scientific), and 1 μL of the cDNA mixture. The thermal cycling process involved initial heating at 95°C for 10 min, and then 50 cycles of 15 s at 95°C and 1 min at 60°C. TaqMan probes and primers for CXCL13 (Hs00757930_m1) and HMBS (Hs00609297_m1) were obtained from Thermo Fisher Scientific. The PCR assay was conducted with the use of the ViiA 7 real‐time PCR system (Thermo Fisher Scientific).

### 2.5 Quantification of Serum CXCL13 and SCC Proteins

Serum CXCL13 protein levels were assessed with the use of the Human CXCL13/BLC/BCA‐1 Quantikine ELISA Kit (R&D Systems, Minneapolis, MN, USA) following the manufacturer's instructions. The serum levels of CXCL13 were measured using a standard curve generated using samples from the control group, and serum SCC antigen levels were quantified using the Alinity SCC・Abbott kit (Abbott Laboratories, Abbott Park, IL, USA), with a cut‐off level set at 1.5 ng/mL.

### 2.6 Spatial Transcriptomics

We analyzed formalin‐fixed paraffin‐embedded (FFPE) primary tumor tissues from two patients with OSCC to carry out transcriptome analysis. Following confirmation of RNA quality (DV200 > 50%) in these tissues, 10‐μm‐thick sections were mounted on the Visium Spatial Gene Expression Slide (10x Genomics, Pleasanton, CA, USA). After obtaining hematoxylin and eosin (H&E)‐stained images by an all‐in‐one microscope BZ‐X810 (Keyence, Osaka, Japan), libraries were constructed using Visium Spatial Gene Expression Reagent Kits for FFPE (10x Genomics) following the manufacturer's instructions. Subsequently, the NovaSeq 6000 system (Illumina, San Diego, CA, USA) was utilized to sequence the libraries. Afterward, the obtained sequencing data and H&E images were analyzed and visualized using Space Ranger v1.3.1 and Loupe Browser v7.0.1, respectively (10x Genomics). Ethical approval was granted by the Ethics Review Board of Ehime University Hospital (Approval Number: 2302007).

### 2.7 Statistical Analysis

The differences between groups were assessed by utilizing the Student's *t*‐test, and those with *p*‐values less than 0.05 were considered statistically significant. The Kaplan–Meier and log‐rank tests were performed to analyze OS and DFS. All statistical examinations were conducted with the use of GraphPad Prism 9.5 software (GraphPad Software, San Diego, CA, USA).

## 3 Results

### 3.1 Novel Biomarker Candidates for OSCC by Microarray

Utilizing Human Genome Survey Arrays, we analyzed the gene expression signatures of 10 primary OSCC and 3 normal oral mucosa tissues [10]. Our findings revealed nine genes exhibiting over 20‐fold higher expression in OSCC tissues compared with that in healthy tissues (Table 1). Among these genes, we focused specifically on CXCL13 because it encodes a soluble secreted protein and is a promising candidate biomarker for OSCC.

**TABLE 1:** Novel biomarker candidates for OSCC identified using microarray analysis.

Gene symbol	Gene name	Fold change	*p*	Cellular component
*MMP3*	Matrix metallopeptidase 3	80.0	0.00068	Extracellular matrix
*MMP1*	Matrix metallopeptidase 1	68.4	0.00104	Extracellular matrix
*MMP11*	Matrix metallopeptidase 11	44.8	0.00104	Extracellular matrix
*MMP10*	Matrix metallopeptidase 10	40.2	0.00379	Extracellular matrix
*CXCL13*	C‐X‐C motif chemokine ligand 13	34.0	0.00104	Soluble fraction, extracellular space
*PTHLH*	Parathyroid hormone‐like hormone	30.6	0.00162	Nucleus, cytoplasm, extracellular space
*HOXC13*	Homeobox C13	24.1	0.00068	Nucleus
*GBP5*	Guanylate binding protein 5	22.3	0.00104	Nucleus, cytoplasm
*CFAP251*	Cilia‐ and flagella‐associated protein 251	20.7	0.00379	Cytoplasm

### 3.2 Expression of CXCL13 mRNA in Primary OSCC Tissues

**FIGURE 1:**
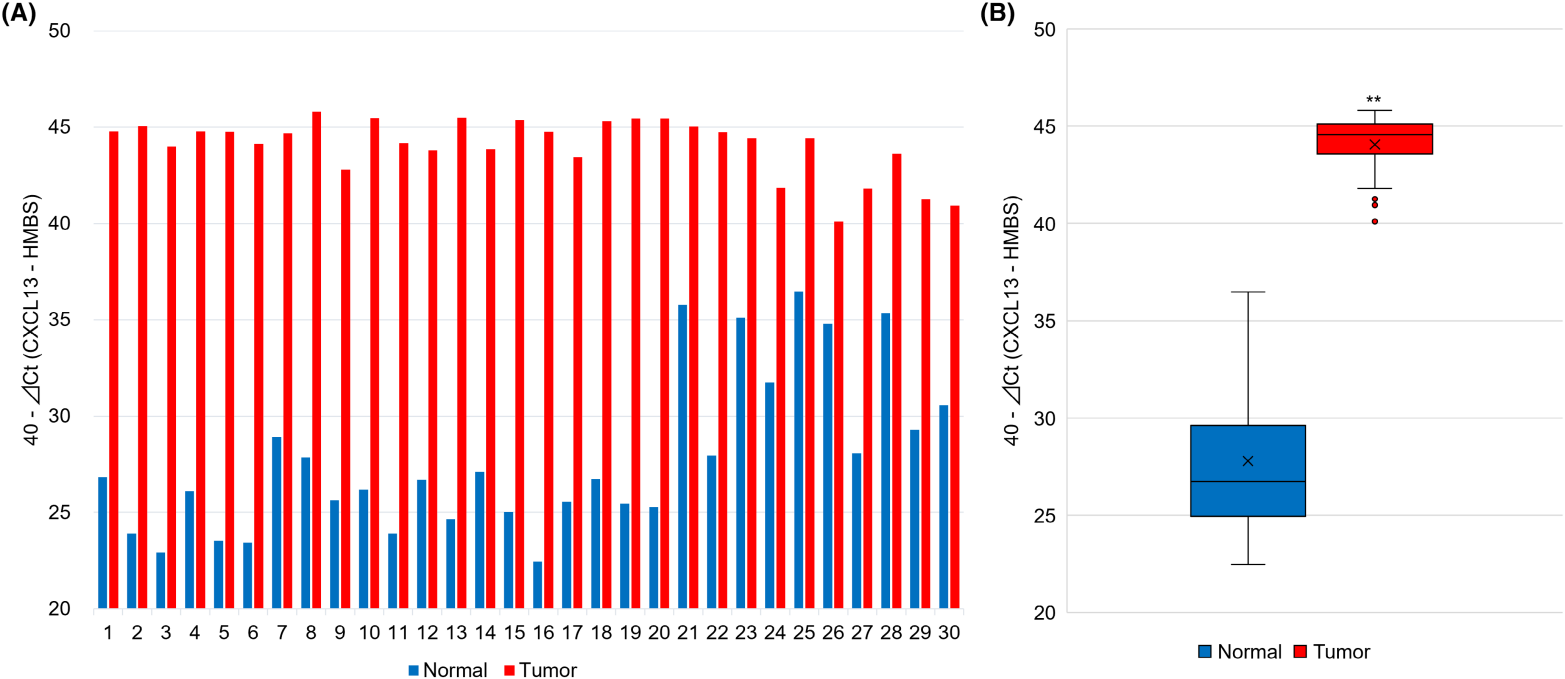
Expression of CXCL13 mRNA in primary OSCC tissues by RT‐qPCR. (A) CXCL13 expression was higher in tumor tissues compared to adjacent normal tissues in all cases. (B) Significant upregulation of CXCL13 mRNA was observed in tumor tissues. **p < 0.01 compared to adjacent normal tissues.

We evaluated CXCL13 mRNA expression in 30 primary OSCC and adjacent healthy tissues using RT‐qPCR. Across all samples, CXCL13 expression levels were consistently higher in the tumor tissues relative to the adjacent healthy tissues (Figure 1A). Furthermore, statistical analysis demonstrated a noticeable increase in the expression levels of CXCL13 mRNA in primary OSCC tissues as compared to adjacent healthy tissues (Figure 1B).

### 3.3 Serum Levels of CXCL13 Protein in Patients With OSCC

**FIGURE 2:**
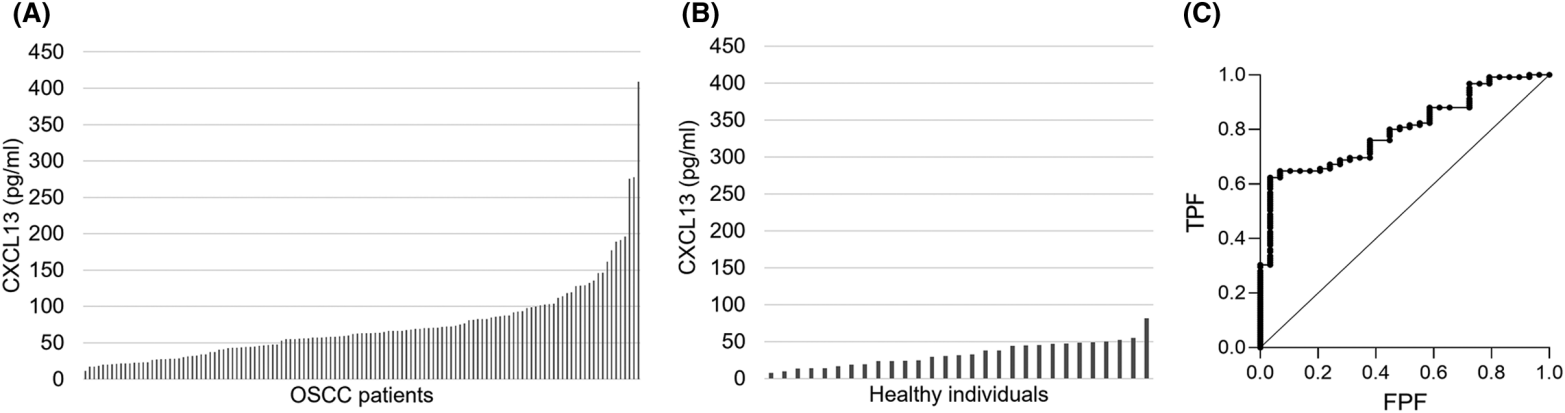
Serum levels of CXCL13 protein in patients with OSCC. (A) The average serum CXCL13 protein in 125 patients with OSCC was 72.8 ρg/mL. (B) The average serum CXCL13 protein in healthy individuals was 33.5 ρg/mL. (C) The reference value determined by the ROC curve for maximizing the TPF/FPF ratio was 53.0 ρg/mL. FPF, false‐positive fraction; TPF, true‐positive fraction.

Serum samples were collected from 125 patients who were diagnosed with OSCC and underwent radical treatment at Ehime University Hospital, as well as from 29 healthy individuals. The level of CXCL13 protein in the serum was assessed by ELISA (Figure 2A,B). By analyzing the receiver operating characteristic (ROC) curve, a reference value for OSCC was established at more than 53.0 ρg/mL (Figure 2C). In healthy individuals, the average serum CXCL13 level was 33.5 ρg/mL, with a specificity of 93.1%. Elevated serum levels of CXCL13 protein were prominently observed in patients with OSCC when contrasted with healthy individuals. In particular, the average serum CXCL13 level in patients with Stage I/II was 66.3 ρg/mL, with a sensitivity of 57.9%. In contrast, in patients with Stage III/IV, the average serum CXCL13 level was 79.4 ρg/mL, with a sensitivity of 69.1%. Importantly, serum CXCL13 protein levels exhibited significant differences between early and advanced OSCC, with values increasing with tumor progression (Table 2).

**TABLE 2:** Serum CXCL13 levels in patients with OSCC.

	Average of serum CXCL13 levels (ρg/mL)	Sensitivity (%)
Healthy individuals (*n* = 29)	33.5	
Patients with OSCC (*n* = 125)	72.8	64.0
Stage I/II (*n* = 57)	66.3	57.9
Stage III/IV (*n* = 68)	79.4	69.1

### 3.4 Diagnostic Sensitivity of Serum CXCL13 and SCC Antigen

CXCL13 protein and SCC antigen levels were quantified using the same serum samples obtained from patients with OSCC. Notably, the sensitivity of serum CXCL13 was markedly higher than that of serum SCC across all stages, as evident from the comparison presented in Table 3.

**TABLE 3:** Diagnostic sensitivity of serum CXCL13 and SCC in patients with OSCC.

OSCC	CXCL13		SCC	
Stage	Average (ρg/mL)	Sensitivity (%)	Average (ng/mL)	Sensitivity (%)
I/II (*n* = 57)	66.3	57.9	1.23	12.1
III/IV (*n* = 68)	79.4	69.1	2.39	48.5

### 3.5 Serum CXCL13 Levels and Clinicopathological Factors in Patients With OSCC

**FIGURE 3:**
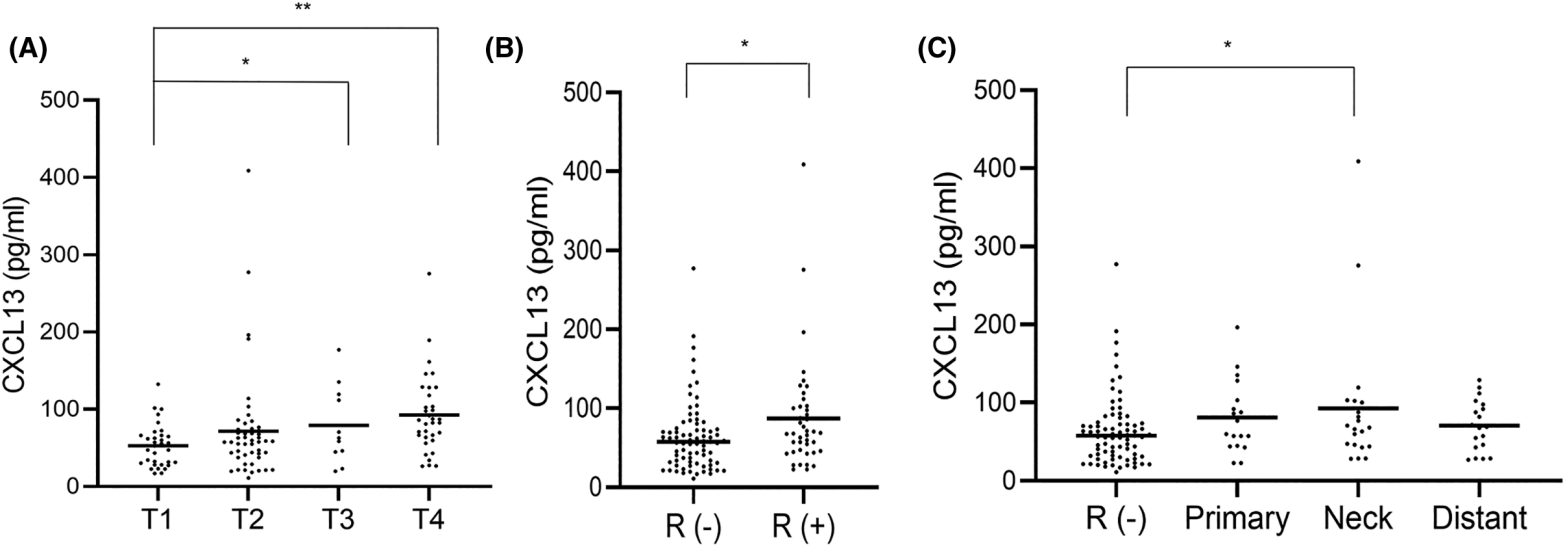
Serum CXCL13 and clinicopathological factors in patients with OSCC. (A) Serum CXCL13 protein levels in T classification. The serum CXCL13 proteins increased in accordance with primary tumor size. The serum CXCL13 levels in T1 were significantly higher than in T3 and T4. (B) The serum CXCL13 proteins in recurrence. The serum levels of CXCL13 proteins in patients with recurrence were significantly elevated. (C) Patients with recurrence were classified into primary recurrence, neck LNM, and distant metastasis (including multiple responses). The serum levels of CXCL13 proteins in neck LNM were significantly elevated. *p < 0.05, **p < 0.01.

Regarding the relationship between serum CXCL13 levels and clinicopathological factors in patients with OSCC, our study revealed a notable increase in serum CXCL13 levels corresponding to the primary tumor size, with a significant elevation observed between T1 and T3, as well as between T1 and T4 (Figure 3A). However, no significant correlation was identified between the N and M classifications (data not shown). Furthermore, we investigated the relationship between serum CXCL13 levels and disease recurrence. Patients experiencing recurrence had significantly higher‐serum CXCL13 levels than patients without recurrence (Figure 3B). The recurrence events were categorized into primary site recurrence, neck LNM, and distant metastasis (with potential overlap). Notably, considerably higher‐serum CXCL13 levels were seen in patients with neck LNM than in those without recurrence (Figure 3C).

### 3.6 Serum CXC13 Levels and Prognosis in Patients With OSCC

**FIGURE 4:**
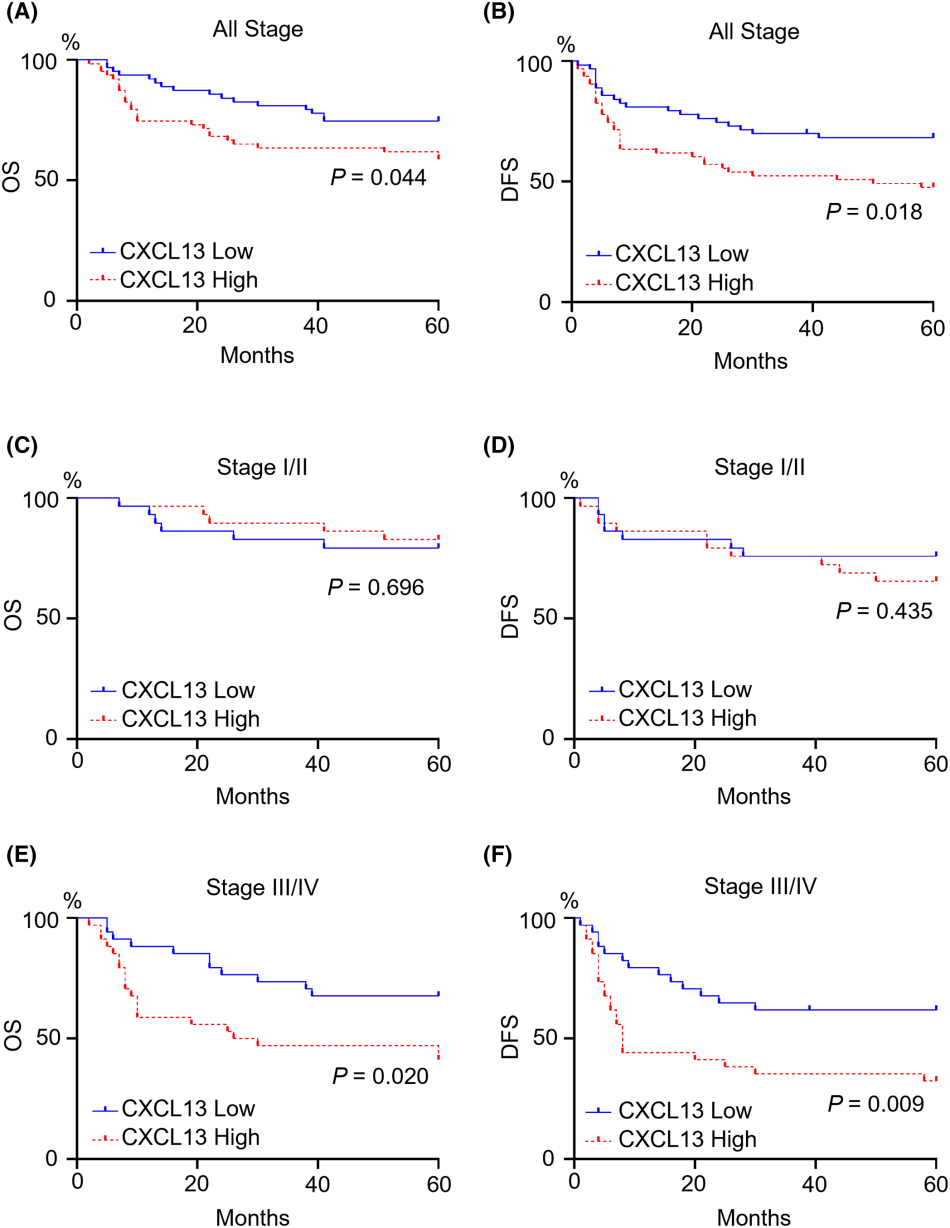
Serum CXCL13 and prognosis in patients with OSCC. A comparison of overall survival (OS) and disease‐free survival (DFS) in patients with OSCC was made among the two groups, which were classified based on the median serum CXCL13 level using the Kaplan–Meier method with a log‐rank test. High expression of serum CXCL13 indicated poor prognosis in both OS (A, *p* = 0.044) and DFS (B, *p* = 0.018) across all stages. In Stage I/II, the groups had no significant difference in OS (C, *p* = 0.696) and DFS (D, *p* = 0.435). In Stage III/IV, both OS (E, *p* = 0.020) and DFS (F, *p* = 0.009) were significantly poorer in patients with high CXCL13 levels.

Patients with OSCC, whose serum CXCL13 levels were assessed, were classified into high‐serum CXCL13 group and low‐serum CXCL13 group by the median values. The Kaplan–Meier method was utilized to carry out a survival analysis. Across all stages, both OS and DFS were considerably worse in the high‐serum CXCL13 group compared to the low‐serum CXCL13 group (Figure 4A,D). Furthermore, patients were divided into early (Stage I/II) and advanced (Stage III/IV) categories for further comparison. In patients with early OSCC, there were no significant differences in both OS and DFS (Figure 4B,E). Conversely, in patients with advanced OSCC, both OS and DFS were notably worse in the high‐serum CXCL13 group compared to the low‐serum CXCL13 group (Figure 4C,F).

### 3.7 Localization of CXCL13 Expression in Primary OSCC Tissues

**FIGURE 5:**
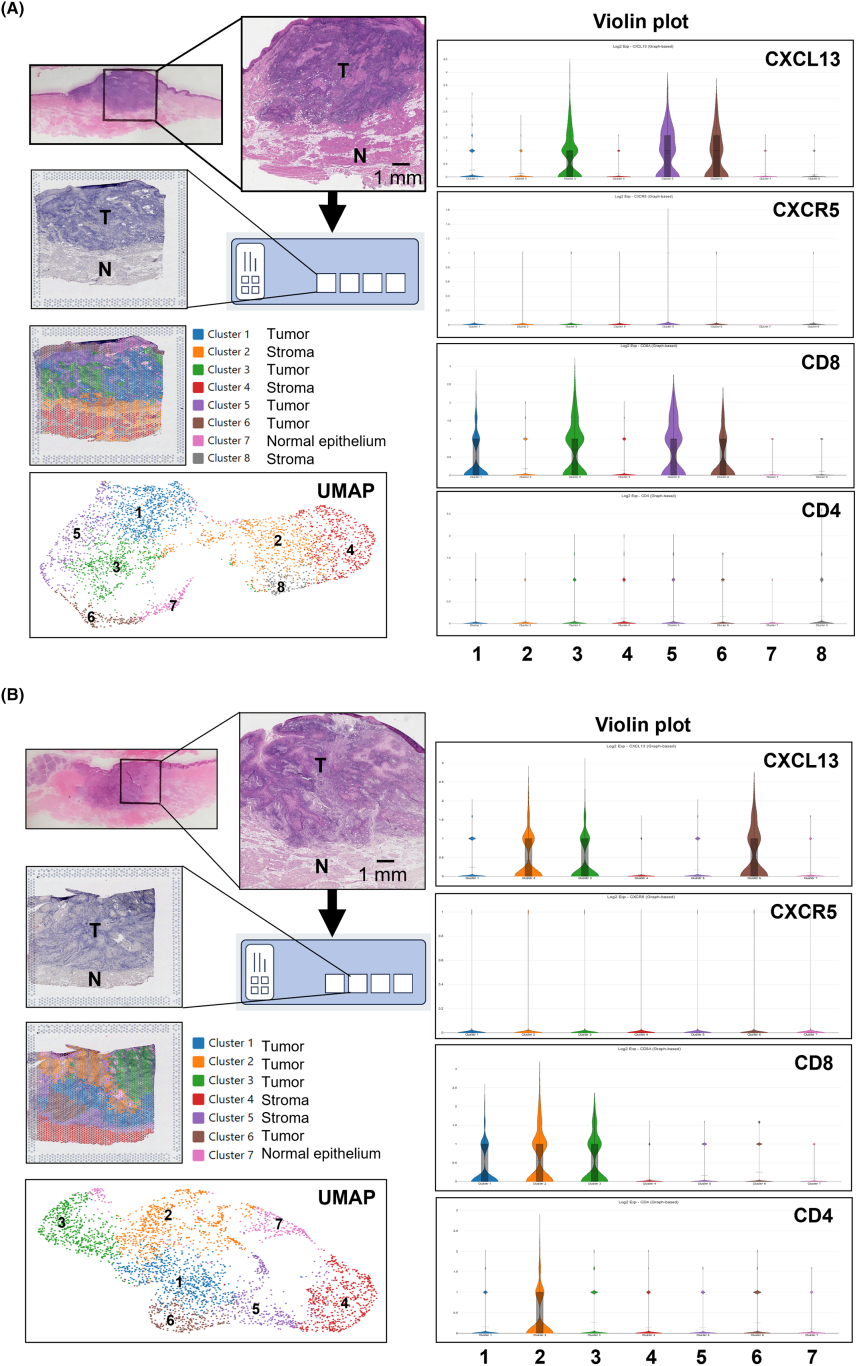
Visium spatial transcriptome analysis using two cases of primary OSCC tissues. (A) Case 1: Primary tongue SCC tissues were classified into eight clusters based on the gene expression profile. Among these, the tumor area comprised four clusters (Cluster 1, 3, 5, and 6). CXCL13 expression was upregulated in three of four clusters within the tumor area. CD8 expression was detected in all tumor clusters. (B) Case 2: In Case 2, primary tongue SCC tissues were classified into seven clusters. The tumor area consisted of four clusters (Clusters 1, 2, 3, and 6). The expression of CXCL13 and CD8 was upregulated in three of four clusters within the tumor area. CD4 expression was detected only in Cluster 2 in the tumor area but not in Case 1.

Finally, we conducted visual spatial transcriptome analysis to elucidate the expression and localization of CXCL13 and its associated molecules in two cases of early OSCC. In both cases, the primary tongue SCC tissues were categorized into eight or seven clusters based on their gene expression profiles. Notably, the tumor area exhibited heterogeneity, comprising four distinct cluster types. While CXCL13 expression was upregulated in three out of four clusters within the tumor area, it was nearly undetectable in the normal epithelium and stroma (Figure 5A,B). Furthermore, we explored the expression of CXCR5, the receptor for CXCL13, along with CD8 and CD4, expressed by CXCL13‐producing T cells. Our findings revealed that CXCR5 expression was markedly low in all tumor and normal clusters. Conversely, CD8 was expressed in almost all tumor clusters but not in normal areas (Figure 5A,B). Additionally, CD4 expression was only detected in one of the four tumor area clusters, which also expressed CXCL13 and CD8 in Case 2 (Figure 5B).

## 4 Discussion

In this study, CXCL13 was identified through a comparative examination of gene expression patterns between primary OSCC and normal oral mucosal tissues using microarray analysis. Additionally, serum levels of CXCL13 were significantly higher in patients with OSCC than in healthy individuals.

Chemokines of the CXC family and their associated receptors play pivotal roles in tumor biology. CXCL13, a chemokine specifically expressed and released by various immune cells like T cells and dendritic cells, has been found to promote the chemotaxis of B cells [13]. CXCL13 is a signaling molecule that binds to its receptor, CXCR5, and is known to be involved in several cancer‐related pathways. These pathways comprise the phosphatidylinositol‐3 kinase (PI3K)/protein kinase B (AKT), mitogen‐activated protein kinase (MEK)/extracellular signal‐regulated kinase (ERK), and PI3K/AKT/mammalian target of rapamycin (mTOR). The presence of CXCL13 in the tumor environment has been suggested to influence cancer cell proliferation and migration [14, 15, 16]. Elevated CXCL13 expression in human malignancies has been associated with various pathways and functions that contribute to tumor progression and immune responses. In prostate cancer, increased CXCL13 expression has been linked to the regulation of infiltration and proliferation through several signaling pathways, including AKT1/2‐cyclin‐dependent kinases 1/2 (CDK1/2)‐CDK inhibitor 1B (CDKN1B), integrin β3‐focal adhesion kinase (FAK)/Src‐paxillin (PXN), AKT‐JUN‐cyclic AMP response‐element binding protein (CREB1), c‐Jun N‐terminal kinase (JNK), and ERK [17, 18]. Similarly, in hepatocellular carcinoma, elevated CXCL13 expression suggests a potential promotion of cancer growth via the Wnt/ß‐catenin pathway [19, 20, 21, 22]. In vitro studies with OSCC cells expressing CXCL13 have demonstrated their potential to infiltrate the jawbone through RANKL stimulation [23]. Likewise, CXCL13 has been implicated in modulating antitumor immune responses. For instance, in ovarian cancer, CD8^+^ cytotoxic T lymphocytes (CTLs) producing CXCL13 have been shown to promote the development of TLS and contribute to antitumor responses [24]. However, CXCL13 can induce B cells to produce the immunosuppressive cytokine IL‐10 in tumor tissues. Similarly, knockout mouse studies have demonstrated a notable decrease in lung metastasis of melanoma in the absence of CXCL13 [25]. In clear cell renal cell carcinoma, T cells secreting CXCL13 infiltrate tumor tissues, expressing exhaustion markers like T‐cell immunoglobulin mucin 3 and programmed cell death protein 1 on their cell surface. This results in decreased levels of antitumor cytokines like tumor necrosis factor α and interferon γ, leading to evasion of antitumor immunity [26]. In OSCC, Chen et al. investigated the functions of CD4^+^ T cells and B cells and revealed through prognosis analysis that patients who have a significant percentage of CD4^+^ CTLs expressing CXCL13 in OSCC tissues tend to have poorer outcomes [27]. Collectively, these findings suggest a relationship between upregulated CXCL13 expression and poor prognosis in various types of cancer.

Notably, the relationship between serum CXCL13 levels and prognosis has been investigated in human malignancies. Sven et al. found that patients with bile duct cancer who had high levels of serum CXCL13 had a significantly lower OS rate than those with low levels [28]. Similarly, Miao et al. reported an association between preoperative serum CXCL13 levels and T classification, pelvic LNM, and pathological grade in penile cancer [29]. Additionally, an increase in serum CXCL13 concentration has been observed in patients with breast cancer who have metastasis compared to those without metastasis [30, 31]. However, no reported association exists between serum CXCL13 and prognosis in OSCC. Here, serum CXCL13 levels were compared between patients with early and advanced OSCC and healthy individuals, revealing markedly higher‐serum CXCL13 levels in patients with OSCC, regardless of stage. Furthermore, the sensitivity of serum CXCL13 was considerably greater at all stages in comparison to routinely used serum SCC antigen levels. Following radical treatment for OSCC, a significant decrease in serum CXCL13 levels was seen in patients at all stages when compared with levels before treatment (Figure S1). This indicates that the serum CXCL13 level is elevated in association with cancer, indicating its potential utility as a novel diagnostic and prognostic marker for OSCC. Although CXCL13 is released as a chemokine associated with the immune response in inflammatory reactions [32], no clear correlations were observed between serum CXCL13 levels and C‐reactive protein, a marker of inflammation (Figure S2). This suggests that serum CXCL13 protein may not necessarily be secreted due to chronic inflammation in cancer tissues. Additionally, higher‐serum CXCL13 levels were noted in patients with delayed neck LNM when compared with patients without recurrence. As controlling neck LNM is critical for determining the prognosis of OSCC, our findings indicate that serum CXCL13 may be utilized as a potential biomarker for detecting occult LNM [33]. However, it is important to recognize that CXCL13 does not act in isolation but rather interacts with multiple signals from cancer cells and surrounding cells, contributing to cancer development [34]. Therefore, relying solely on the individual test value of CXCL13 to assess cancer risk may be inadequate. A comprehensive evaluation involving a combination of biomarkers and consideration of clinical symptoms may be necessary for more accurate cancer risk assessment.

In this study, we found that in advanced OSCC, both OS and DFS were notably worse in patients with high levels of serum CXCL13 compared to those with low levels of serum CXCL13. This suggests a potential involvement of this chemokine in OSCC progression, possibly promoting tumor growth and metastasis, thus leading to a poorer prognosis. However, it is essential to note that our study only establishes an association between serum CXCL13 levels in OSCC and poor prognosis, and its functional role remains unclear. Therefore, in future investigations, we plan to delve deeper into the expression patterns of CXCL13 in OSCC tissues, decipher its functional roles, establish its correlation with prognosis, and investigate its potential as a therapeutic target. By doing so, we hope to further explore the relationship between OSCC and CXCL13, shedding light on the specific roles CXCL13 plays in OSCC pathology and establishing its significance in the diagnosis and treatment of patients with OSCC.

In conclusion, our study identified CXCL13 as a consistently elevated and promising biomarker of primary OSCC. Elevated serum CXCL13 levels showed significant diagnostic potential, correlating with tumor characteristics. Additionally, the clear relationship between high‐serum CXCL13 level and poor prognosis in patients with OSCC with advanced stage was particularly noteworthy, emphasizing its crucial role in prognostic assessment. These findings underscore CXCL13 as a potentially useful novel biomarker with diagnostic and prognostic significance for OSCC, paving the way for further clinical investigation.

## Author Contributions

**Shin Tojo:** formal analysis (equal), investigation (equal), resources (equal), writing – original draft (equal). **Koh‐ichi Nakashiro:** conceptualization (lead), data curation (lead), funding acquisition (lead), methodology (lead), project administration (lead), software (lead), validation (lead), visualization (lead), writing – review and editing (lead). **Nobuyuki Kuribayashi:** formal analysis (equal), investigation (equal), resources (equal), writing – original draft (equal). **Daisuke Uchida:** supervision (lead), writing – review and editing (supporting).

## Ethics Statement

The research was carried out in accordance with the principles outlined in the Declaration of Helsinki and was authorized by the Institutional Review Board at Ehime University Hospital for studies involving humans (Approval Numbers: 1002011 and 2302007).

## Consent

All of the participants in the study provided their informed consent.

## Conflicts of Interest

The authors declare no conflicts of interest.

## Supporting information


Figure S1.

Figure S2.

Table S1.

## Data Availability

The results presented in this research are supported by data available at https://www.ncbi.nlm.nih.gov/geo/query/acc.cgi?acc=GSE36090, released on September 30, 2012.
