# Supplementary figures and images for: Serum CXCL13 as a Novel Biomarker in Oral Squamous Cell Carcinoma
Source: Cancer Med. 2024 Sep 30;13(18):e70263. doi: 10.1002/cam4.70263 (PMC11440027; doi:10.1002/cam4.70263)

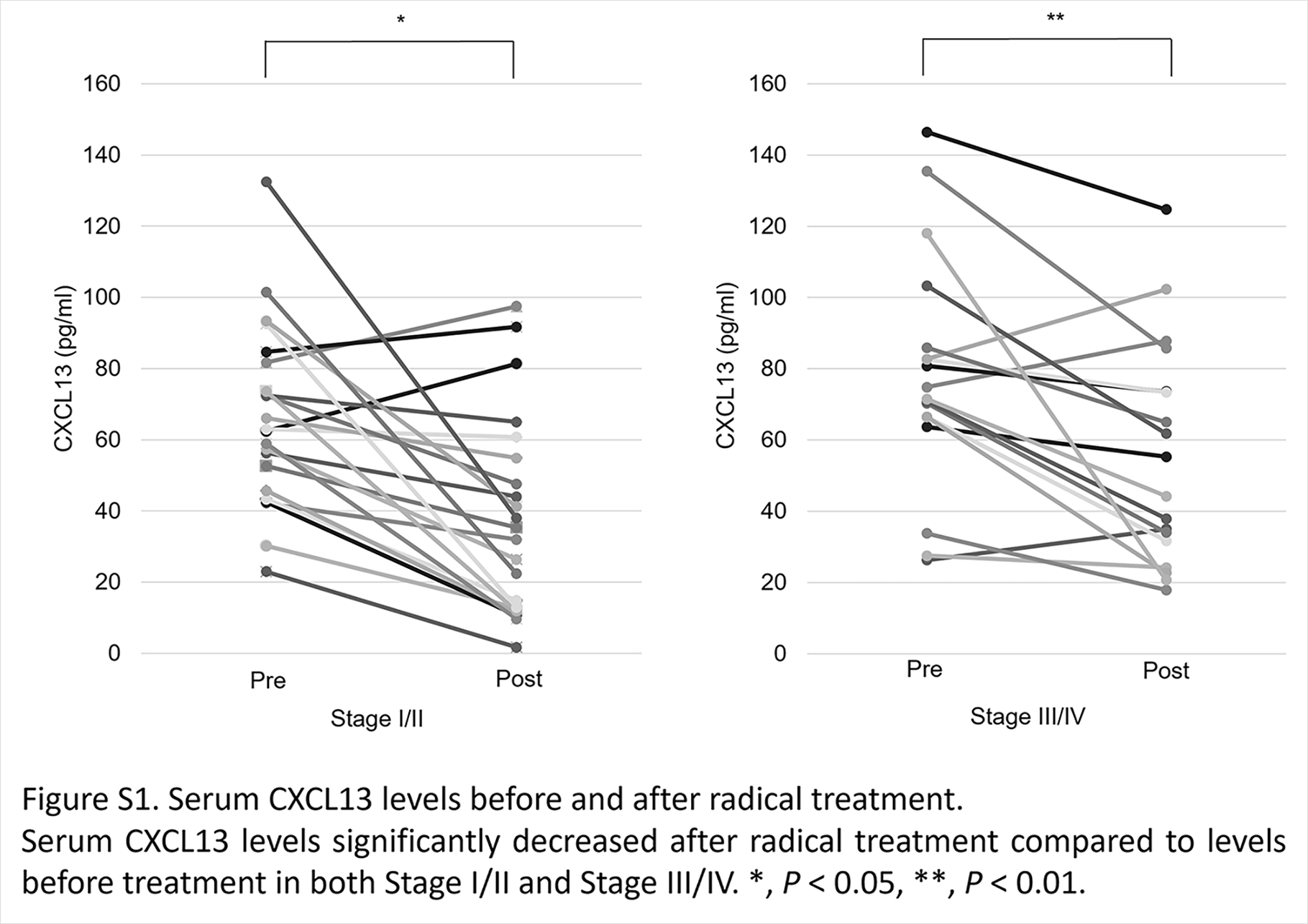

Supplement: Supplementary file 1 — Figure S1. [file CAM4-13-e70263-s003.tif]

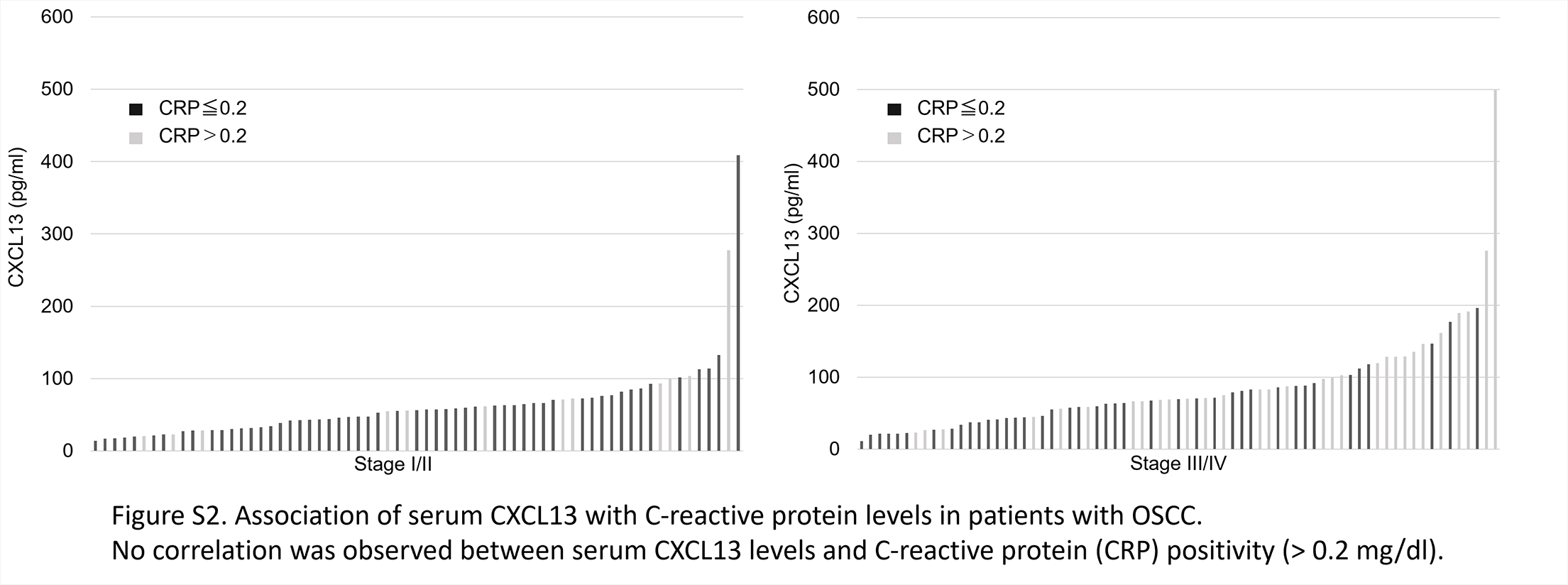

Supplement: Supplementary file 2 — Figure S2. [file CAM4-13-e70263-s001.tif]
