# Supplementary material for: Serum CXCL13 as a Novel Biomarker in Oral Squamous Cell Carcinoma
Source: Cancer Med. 2024 Sep 30;13(18):e70263. doi: 10.1002/cam4.70263 (PMC11440027; doi:10.1002/cam4.70263)
Supplement: Supplementary file 3 — Table S1. [file CAM4-13-e70263-s002.docx]

Table S1. Baseline characteristics of 125 patients with OSCC

| **Clinicopathological factors** | | **Number of patients (％)** |
| --- | --- | --- |
| Age | < 65 | 44 (35.2) |
|  | ≤ 65 to < 75 | 38 (30.4) |
|  | ≤ 75 | 43 (34.4) |
| Gender | Male | 75 (60.0) |
|  | Female | 50 (40.0) |
| Primary site | Tongue | 51 (40.8) |
|  | Lower gingiva | 40 (32.0) |
|  | Upper gingiva | 17 (13.6) |
|  | Floor of mouth | 9 (7.2) |
|  | Buccal mucosa | 8 (6.4) |
| Stage | I | 30 (24.0) |
|  | II | 27 (21.6) |
|  | III | 14 (11.2) |
|  | IVA | 44 (35.2) |
|  | IVB | 9 (7.2) |
|  | IVC | 1 (0.8) |
| T | 1 | 33 (26.4) |
|  | 2 | 47 (37.6) |
|  | 3 | 11 (8.8) |
|  | 4a | 30 (24.0) |
|  | 4b | 4 (3.2) |
| N | 0 | 76 (60.8) |
|  | 1 | 12(9.6) |
|  | 2b | 20 (16.0) |
|  | 2c | 11 (8.8) |
|  | 3b | 6 (4.8) |
| M | 0 | 124 (99.2) |
|  | 1 | 1 (0.8) |
| Differentiation | G1 | 81 (64.8) |
|  | G2 | 29 (23.2) |
|  | G3 | 15 (12.0) |
| Recurrence | － | 79 (63.2) |
|  | ＋ | 46 (36.8) |
